# A Hexadecanuclear Cobalt-Added Tungstogermanate Containing Counter Cobalt Hydrates: Synthesis, Structure and Photocatalytic Properties

**DOI:** 10.3390/nano13132009

**Published:** 2023-07-05

**Authors:** Qing Zhao, Xuyan Li, Yu Wang, Hongjin Lv, Guoyu Yang

**Affiliations:** MOE Key Laboratory of Cluster Science, School of Chemistry and Chemical Engineering, Beijing Institute of Technology, Beijing 102488, China; zhaoqing980121@163.com (Q.Z.); lxy_bitchem@163.com (X.L.); yuwang0414@outlook.com (Y.W.)

**Keywords:** hydrothermal synthesis, cobalt-added polyoxometalate, photocatalysis, hydrogen evolution

## Abstract

The synthesis and exploration of the properties of structurally-new polyoxometalates (POMs) has been attracting considerable research interest. In this work, a hexadecanuclear cobalt-added tungstogermanate, H_31_(NH_4_)_5_Na_16_{Co^Ⅲ^(H_2_O)_6_}_4_{[Co^Ⅱ^_4_(μ_3_-OH)_3_(PO_4_)]_4_(A-*α*-GeW_9_O_34_)_4_}_2_·23-H_2_O (**1**), was synthesized under hydrothermal conditions and characterized by various techniques. Compound **1** can effectively drive the heterogeneous photocatalytic hydrogen evolution reaction in the presence of [Ir(ppy)_2_(dtbbpy)][PF_6_] as the photosensitizer, with triethanolamine (TEOA) and N-Hydroxy succinimide (NHS) used as the dual sacrificial reagents. Control experiments revealed the important role of NHS in enhancing the hydrogen-evolution activities. Under optimal catalytic conditions, a hydrogen yield of 54.21 μmol was achieved after 10-h photocatalysis, corresponding to a hydrogen evolution rate of 1807.07 μmol·g^−1^·h^−1^. Stability studies demonstrated that catalyst **1** can be isolated and reused for three successive photocatalytic cycles with negligible decline of the H_2_ yield, indicating the stability and recycling robustness of catalyst **1**.

## 1. Introduction

Polyoxometalates (POMs), a class of nano-sized metal-oxo clusters, are formed through the condensation of metalates in their high oxidation states (e.g., Mo^VI^, W^VI^, V^V^, Nb^V^, Ta^V^) [1,2], which usually exhibit some advantages: tunable molecular structures, reversible multi-electron-storing abilities, and excellent redox properties [3,4]. The explicit structures of POMs can be easily determined by modern crystallographic characterization techniques, making it convenient to investigate their physicochemical properties at the atomic level [5,6]. To date, POMs have been widely used in the fields of catalysis [7,8,9], electrochemistry [10,11], magnetism [12,13], and pharmaceutical chemistry [14]. Typically, lacunary Keggin-type POMs (e.g., {XW_9_O_34_}, X = Si, Ge, P, etc.) can be generated by removing one or several {WO_6_}s from the plenary {XW_12_O_40_} (X = Si, Ge, P, etc.) structures. The lacunary tungsten-oxo clusters can act as multidentate inorganic ligands, incorporating transition metal ions to form transition metal-added POMs (TMAPs) [13,15]. By following the lacunary-directing, synthetic strategy, numerous TMAPs have been reported [15,16,17,18,19,20,21,22,23,24]. TMAPs have been comprehensively studied due to their integrated advantages, including added transition metal ions and lacunary POM ligands, which result in good structural stability and interesting catalytic properties [20,21].

Among various TMAPs, the synthesis and application of high-nuclearity, cobalt-added polyoxometalates (CoAPs) remain one of the most attractive research directions for global researchers. By controlling reaction conditions, lacunary tungsten-oxo clusters can be made to undergo diverse configuration changes. Different lacunary tungsten-oxo clusters possess contrasting coordination abilities, which can therefore induce cobalt ions to aggregate in various forms. Some representative high-nuclearity CoAPs include [{(B-α-PW_9_O_34_)Co_3_(OH)(H_2_O)_2_(O_3_PC(O)(C_3_H_6_NH_3_)PO_3_)}_2_Co]^14−^ [25], [Co_9_(OH)_3_(H_2_O)_6_(PO_4_)_2_(B-*α*-PW_9_O_34_)_3_]^21−^ [26], [Co_6_(H_2_O)_30_{Co_9_Cl_2_(OH)_3_(H_2_O)_9_(*β*-SiW_8_O_31_)_3_}]^5−^ [27], [{Co_4_(OH)_3_PO_4_}_4−_ (PW_9_O_34_)_4_]^28−^ [12,20], and [Co_21_(H_2_O)_4_(OH)_12_(SiW_10_O_37_)_6_]^30−^ [28]. In these reported CoAPs, the high-nuclearity aggregation of cobalt-oxo clusters can be achieved with the aid of some organic/inorganic linkers. With respect to the synthetic approaches, most of these reported CoAPs have been synthesized using the conventional aqueous solution-based method, which usually requires good solubility of starting materials in water. In contrast, the emerging hydro(solvo)thermal synthetic approach could be more applicable to synthetic systems even though the solubility of starting reagents is relatively poor. In addition, the high-temperature and -pressure environment associated with hydro(solvo)thermal reactions is beneficial for the formation of some structurally-new POM intermediates, which contrasts with the thermodynamically dominant products synthesized by the conventional solution-based method [1,2,3].

Cobalt-based complexes have exhibited interesting catalytic applications, especially, given the increasing concerns about the energy crisis and environmental problems, in solar-driven water splitting [29,30]. Due to the complexity of total water splitting reactions, they have often been studied in separate half-parts: oxygen evolution reactions (OERs) and hydrogen evolution reactions (HERs) [31]. To date, the CoAPs have been widely investigated as water oxidation catalysts in both photo-/electrochemical systems [9,20,25,32,33,34,35,36,37,38]. The well-defined structures of CoAPs are beneficial for comprehensive studies on the relationship between the catalytic mechanism and structure properties. In contrast, very few works have been published that introduce CoAPs as catalysts in visible-light-driven water-reduction reactions [39,40,41], although many TMAPs were reported as the multi-electron-transfer catalysts for HERs [7,42,43,44,45]. In this regard, the continuous design and photocatalytic HER exploration of novel, high-nuclearity CoAPs remains a critical and interesting research direction.

Herein, we have successfully synthesized and systematically characterized water-insoluble and stable CoAPs, H_31_(NH_4_)_5_Na_16_{Co^Ⅲ^(H_2_O)_6_}_4_{[Co^Ⅱ^_4_(μ_3_-OH)_3_(PO_4_)]_4_(A-*α*-GeW_9_O_34_)_4_}_2_·23H_2_O (**1**), under hydrothermal conditions by introducing {GeW_9_O_34_} as the multidentate ligand and phosphate as a bridging linker to the central cobalt-oxo clusters. Compound **1** was investigated as a stable and recyclable heterogeneous catalyst to drive the photocatalytic HER. In the photocatalytic system, dual sacrificial reagents were first applied for improved performance.

## 2. Experiments Section

### 2.1. General Procedure

The chemicals utilized in this work are commercially obtained and used without further purification. The tri-vacant POMs K_8_Na_2_[A-α-GeW_9_O_34_]·25H_2_O is prepared according to the previous report [46,47]. The basic characterization instruments and methods are as follows: (1) A Bruker D8 Advance X-ray diffractometer (Bruker, Karlsruhe, Germany) equipped with Cu Kα radiation (λ = 1.54056 Å) scanning from 5 to 50° was used to determine the powder X-ray diffraction (PXRD) patterns of **1**. (2) The Nicolet iS10 FT-IR spectrometer (Thermo Fisher Scientific, Waltham, MA, USA) was used for IR spectrum (400–4000 cm^−1^) measurements with KBr pellets. (3) The optical gap of **1** was measured and estimated from UV-Vis diffuse reflectance spectra (200–800 nm) using a Shimadzu UV3600 spectrometer (Shimadzu, Kyoto, Japan). (4) Thermal stability was confirmed by thermogravimetric analyses using a Mettler Toledo TGA/DSC 1100 analyzer (Mettler Toledo, Zurich, Switzerland). The testing temperature ranges from 25 to 1000 °C in air at a heating rate of 10 °C·h^–1^. (5) Inductively coupled plasma mass spectrometry was measured on an ICP-OES (iCAP 7400, Thermo, Waltham, MA, USA) to complete the elemental analysis and post-catalytic characterization.

### 2.2. Synthesis

A mixture of K_8_Na_2_[A-α-GeW_9_O_34_]·25H_2_O (0.30 g, 0.097 mmol), Co(NO_3_)_2_·6H_2_O (0.18 g, 0.62 mmol), NH_4_Cl (0.20 g, 3.73 mmol), and Na_2_HPO_4_·12H_2_O (0.10 g, 0.28 mmol) was introduced into 5 mL of deionized water. After stirring for 30 min, the pH of the solution was regulated to 9.50 by adding 4M of NaOH solution. The resulting purple-red turbid mixture was transferred into a 25 mL, Teflon-lined, stainless-steel autoclave and kept at 100 °C for 3 days. Pink spindle crystals were obtained after cooling the solution to room temperature. The crystals were further purified by washing and sonicating with deionized water. (Yield: 21%, based on K_8_Na_2_[A-α-GeW_9_O_34_]·25H_2_O). IR (KBr, cm^−1^): 3413 (vs), 1633 (vs), 1400 (vs), 1079 (w), 933 (vs), 869 (s), 806 (vs), 688 (vs). Elemental analysis (calcd) for compound **1** (wt %): Co 9.64 (9.30), Ge 3.01 (2.51), Na 1.90 (1.62), P 1.21 (1.09), W 58.21 (57.82).

### 2.3. X-ray Crystallography

A translucent pink crystal of **1** was sealed into a glass tube with Vaseline to collect the diffraction data by a Gemini A Ultra CCD diffractometer equipped with graphite monochromated Mo K*α* (λ = 0.71073 Å) and radiation of 296(2) K. The structure was solved using the direct method and refined using full-matrix least-squares, which were fit using the F^2^ method in Olex2 [48] and the SHELX-2008 program package [49,50]. The contribution of the disordered water molecules to the diffraction data of **1** was examined using the SQUEEZE method in PLATON [51]. The crystalline water molecules cannot be fully identified by refinement (Table 1). The number of water molecules was quantified based on the TGA data shown in Figure S1, where 23 lattice water molecules exist in the structure. To obey the charge balance motif, 31 protons were added. The elemental compositions of compound **1** were further characterized by ICP-OES tests, which correlate with the single-crystal measurement results. The CCDC number of **1** is deposited as 2262758. Further data can be obtained from http://www.ccdc.cam.ac.uk/, accessed on 10 June 2023.

### 2.4. Photocatalytic Water Reduction Tests

The whole photocatalytic reaction system was comprised of acetonitrile/N,N-dimethylformamide (*v*/*v* = 1:3) as the mixed solvent, [Ir(ppy)_2_(dtbbpy)][PF_6_] as the photosensitizer, TEOA and NHS as the dual electron sacrificial reagents, **1** as the catalyst, and distilled water as the proton donor. The above components were transferred and sealed into the photoreactor. The reactor was deaerated by CH_4_/Ar (*v*/*v* = 4:1, CH_4_ as the internal standard substance) in a mixed atmosphere for ten minutes, and then transferred to a PCX-50C multi-channel photocatalytic reactor equipped with a white LED light plate (400–800 nm, electric power: 10 W) for photocatalytic reaction. During the photocatalysis, the reaction solution was continuously stirred at 400 rpm. The reaction temperature was kept at 25 °C by a water-circulating system. At an interval of two hours, 125 μL mix gas was extracted and qualitatively and quantitatively analyzed by a GC-979011 gas chromatograph analyzer.

## 3. Results and Discussion

### 3.1. Structure of Compound ***1***

**Figure 1:**
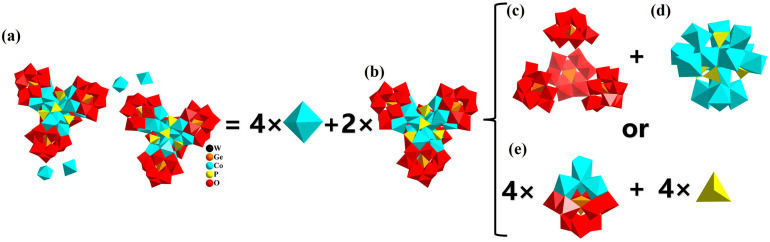
The polyhedron diagrams of (**a**) polyoxoanion and counter cations {Co(H_2_O)_6_} in **1**; (**b**) {[Co_4_(μ_3_-OH)_3_(PO_4_)]_4_(A-α-GeW_9_O_34_)_4_}; (**c**) four {A-α-GeW_9_O_34_}; (**d**) {Co_16_(μ_3_-OH)_12_O_36_(PO_4_)_4_}; (**e**) {Co_4_GeW_9_O_34_}. Color codes: WO_6_, red; GeO_4_, orange; CoO_6_, turquoise; PO_4_, yellow.

**1** crystallizes in the triclinic crystal system, *P*-1 space group. The structure consists of two hexadecanuclear cobalt-added tetrameric tungsten-oxo clusters {[Co_4_(μ_3_-OH)_3_(PO_4_)]_4−_ (A-α-GeW_9_O_34_)_4_} [20,52], four free {Co(H_2_O)_6_} (Figure 1a,b and Figure S2), 16 Na^+^, 5 NH_4_^+^, 31 H^+^ as counter cations, and 23 crystalline water molecules. In the polyoxoanion of **1**, four {A-α-GeW_9_ O_34_} POM ligands are located at the four vertices of a tetrahedron and further anchored to the {Co_16_(μ_3_-OH)_12_O_36_(PO_4_)_4_} cluster through μ_2_-O, μ_3_-O, and μ_4_-O atoms (Figure 1c,d). In another image, six vacant sites of each {A-α-GeW_9_O_34_} induce a defective-cubane-type tetranuclear cobalt-oxo cluster {Co_4_O_3_}, which gives rise to the {Co_4_GeW_9_}. Four identical {Co_4_GeW_9_} moieties are connected by four {PO_4_} linkers and four μ_3_-O atoms (Figure 1e).

All cobalt ions in the polyoxoanion of **1** exist in a hexacoordinated octahedron mode and are chemically present in bivalent forms as determined by the Bond Valence Sum (BVS) calculations [53]. The bond lengths of Co-O vary from 1.98 to 2.27 Å. For the four dissociated {Co(H_2_O)_6_}, the Co-O lengths are in the range of 1.85–2.03 Å. The BVS values of the four cobalt ions are 2.92, 2.89, 2.79, and 2.90, illustrating the existence of Co^Ⅲ^ in the free cobalt hydrates. According to the Bond Valence Sum (BVS) calculation results [53], the chemical valance of W, Ge, and P is +6, +4, and +5, respectively. The oxygen in polyoxoanion **1** has three existing forms including O^2−^, OH, and H_2_O, as revealed by the structural analyses and BVS calculations.

**Figure 2:**
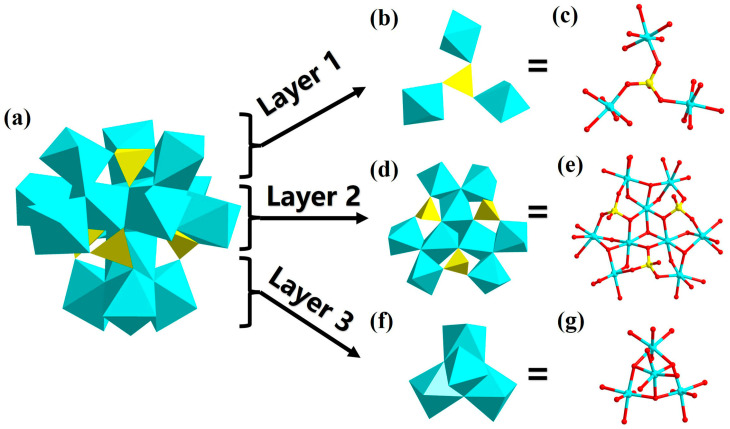
(**a**) Polyhedron diagram of {Co_16_(μ_3_-OH)_12_O_36_(PO_4_)_4_} from the front view. The top view of (**b**) polyhedron diagram of {Co_3_(μ_3_-OH)_6_O_9_(PO_4_)}; (**c**) ball and stick diagram of {Co_3_(μ_3_-OH)_6_O_9_(PO_4_)}; (**d**) polyhedron diagram of {Co_9_(μ_3_-OH)_9_O_19_(PO_4_)_3_}; (**e**) ball and stick diagram of {Co_9_(μ_3_-OH)_9_O_19_-(PO_4_)_3_}; (**f**) polyhedron diagram of {Co_4_(μ_3_-OH)_3_O_14_}; (**g**) ball and stick diagram of {Co_4_(μ_3_-OH)_3_O_14_}. Color codes: CoO_6_, turquoise; PO_4_, yellow; Co atom, turquoise; P atom, yellow; O atom, red.

The hexadecanuclear cobalt-oxo cluster {Co_16_(μ_3_-OH)_12_O_36_(PO_4_)_4_} can be structurally illustrated as three layers (Figure 2a). The first layer is {Co_3_(μ_3_-OH)_6_O_9_(PO_4_)} (Figure 2b,c), where a {PO_4_} linker is located in the center, with three hexacoordinated {Co(μ_3_-OH)_2_O_4_} links to {PO_4_} via three μ_2_-O atoms. The residual μ_4_-O of {PO_4_} is connected to the second layer that is a tri-phosphate-modified clover-like nonanuclear cobalt-oxo cluster {Co_9_(μ_3_-OH)_9_O_19_(PO_4_)_3_} (Figure 2d,e). Three hexacoordinated Co^2+^ are linked together by edge-sharing modes, creating one of the three “leaves” of the “clover”. The three “leaves” are held together by one μ_4_-O atom. Three {PO_4_} linkers are located in the same plane and fixed to the gap between the three “leaves” of the {Co_9_(μ_3_-OH)_9_O_19_(PO_4_)_3_}, connected by P-μ_2_-O-Co, P-μ_3_-O-Co, and P-μ_4_-O-Co linkages. Each of the three {PO_4_}s in the second layer can share a μ_2_-O to connect with the pedestal position Co of the third layer. The third layer is {Co_4_(μ_3_-OH)_3_O_14_} (Figure 2f,g), which is the traditional defective cubane-like configuration tetranuclear cobalt-oxo cluster. The vertex position Co of the third layer was linked to the center of the second layer through an edge-sharing mode.

**Figure 3:**
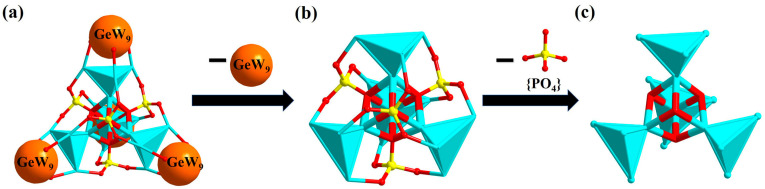
The simplified arrangement diagrams of (**a**) the polyoxoanion in **1**; (**b**) {Co_16_(μ_3_-OH)_12_-O_36_(PO_4_)_4_}; (**c**) {Co_16_(μ_3_-OH)_12_O_52_}. Color codes: GeW_9_, orange ball; Co_4_, turquoise tetrahedron; Co atom, turquoise; P atom, yellow; O atom, red.

In the polyoxoanion **1**, {[Co_4_(μ_3_-OH)_3_(PO_4_)]_4_(A-α-GeW_9_O_34_)_4_} can also be simplified by omitting sectional oxygen ions from {A-α-GeW_9_O_34_} (Figure 3a). Four Co^Ⅱ^ in each {Co_4_(μ_3_-OH)_3_O_14_} can form a {Co_4_} tetrahedron. The four Cos at the vertex position of four identical {Co_4_} tetrahedrons and four μ_4_-O atoms of {PO_4_} linkers can construct a {Co_4_O_4_} cubane in the center of {[Co^Ⅱ^_4_(μ_3_-OH)_3_(PO_4_)]_4_(A-α-GeW_9_O_34_)_4_} (Figure 3b,c).

**Figure 4:**
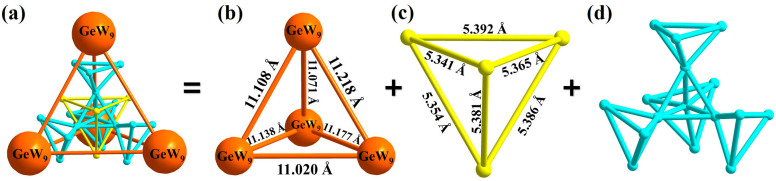
The simplified arrangement diagrams of (**a**) the polyoxoanion in **1**; (**b**) four {A-α-GeW_9_O_34_}; (**c**) four {PO_4_}; (**d**) {Co_16_(μ_3_-OH)_12_O_52_}. Color codes: GeW_9_ orange; Co atom, turquoise; P atom, yellow.

From another view, {[Co_4_(μ_3_-OH)_3_(PO_4_)]_4_(A-α-GeW_9_O_34_)_4_} can also be presented as a tri-nested structure by simplifying {A-α-GeW_9_O_34_} as orange balls, cobalt ions as pink balls, and {PO_4_}s as yellow balls (Figure 4a). The four {A-α-GeW_9_O_34_} are located in the vertices of a tetrahedron, and the distance between each {A-α-GeW_9_O_34_} varies from 11.020 to 11.218 Å (Figure 4b). The arrangement of the four {PO_4_} linkers also shows a tetrahedron configuration. The distances between each {PO_4_} are in the range of 5.341–5.392 Å (Figure 4c). In the simplified {Co_16_(μ_3_-OH)_12_O_36_(PO_4_)_4_}, vertices of the four external {Co_4_} tetrahedrons can form an internal {Co_4_} tetrahedron. The distances between each Co^Ⅱ^ are in the range of 3.017–3.811 Å (Figure 4d). The packing mode of **1** in the *bc* plane is shown in Figure S3.

### 3.2. FT-IR Measurement

As illustrated in Figure S4, the FT-IR spectrum of **1** involves four major characteristic vibrational groups. First, the wide peaks in 3423–3203 cm^−1^ and 1633 cm^−1^ can be attributed to the existence of lattice and coordination water in **1**. The distinct vibrational peak observed at ~1400 cm^−1^ could be attributed to the existence of NH_4_^+^ counter cations. In the fingerprint region, the absorption peak centered at 1079 cm^–1^ is ascribed to the ν(P–O) stretching vibration mode. The typical vibration peaks of the tri-vacant {A-α-GeW_9_O_34_} precursor (e.g., ν(Ge-O) ν(W-Ot), ν(W-Ob), and ν(W-Oc)) are observed at 933 (vs), 869 (vs), 806 (vs), and 688 cm^−1^, respectively.

### 3.3. Powder X-ray Diffraction Patterns

To confirm the phase purity of **1**, it is noted that there is agreement between the experimental PXRD pattern and the simulated pattern from the single-crystal X-ray diffraction data (Figure S5). The modest shift in peak intensity may be attributed to a change in the favored orientation of the powder sample during the PXRD data-collecting process.

### 3.4. UV-Vis Diffuse Reflectance Test

Compound **1** exhibited strong absorption in the range of 200–800 nm. The wide absorption peak at 200–400 nm corresponds to the O-to-W charge transfer, while the absorption of 500–600 nm is attributed to the d-d transitions of Co centers. The obtained absorbance-wavelength data is further processed according to the Kubelka-Munk formula α/S = (1 − R)^2^/(2R) (R = reflection coefficient, α = absorption factor, S = scattering factor) to make the α/S-Energy plot, and the tangent plot is made in the near-linear range of the obtained curve; the intersection point with α/S = 0 is the optical band gap value. As shown in Figure S6, the optical gap of compound **1** is estimated as 2.65 eV.

### 3.5. Photocatalytic Hydrogen Generation

**Figure 5:**
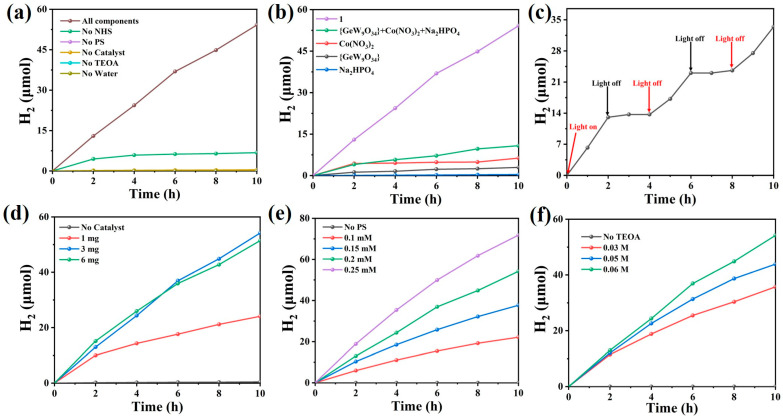
(**a**) A series of blank experiments and the optimal reaction condition test of HER; (**b**) photocatalytic H_2_ evolution with different catalysts; (**c**) effect of the visible light towards the HER. Photocatalytic H_2_ evolution with different amounts of (**d**) **1**; (**e**) photosensitizer; (**f**) TEOA. (Reaction conditions: 10 W white LED light, **1** (1, 3, 5 mg), TEOA (0.03, 0.050, 0.060 M), NHS (0.153 mM), H_2_O (2 M), [Ir(ppy)_2_(dtbbpy)][PF_6_] (0.1, 0.15, 0.2, 0.25 mM), CH_3_CN/DMF (1/3) as mixed solvent, the total reaction volume of 6 mL, stirring speed 400 rpm, at room temperature).

Given the poor solubility of **1** in water, N,N-dimethylformamide, or acetonitrile, **1** was employed as a heterogeneous catalyst in the visible-light-driven hydrogen generation reaction. Various control experiments confirmed that the existence of the catalyst, photosensitizer, and sacrificial agent was indispensable for efficient photocatalysis (Figure 5a, Table S1). It is noted that the very small amount of hydrogen (0.42 μmol) was produced from the control experiment containing Ir photosensitizer and TEOA sacrificial donor, which is commonly observed in the literature [42,43,44,54]. Commonly, one type of electron donor is used in photocatalytic HER systems. Herein, when TEOA was used as the only sacrificial agent, only 6.81 μmol of H_2_ evolved. In 2019, Mialane’s group reported a tetradecanuclear nickel-added tungsten-oxo cluster [(A-α-SiW_9_O_34_)Ni_14_(AleH)_5_(Ale)_2_(H_2_O)_11_-(OH)_7_]^12−^ (Ale = alendronate), which can act as a heterogeneous catalyst to drive the photocatalytic hydrogen evolution reaction in the presence of TEOA, 1-benzyl-1,4-dihydronicotinamide (BNAH), and [Ir(ppy)_2_(dtbbpy)][PF_6_]. Therein, BNAH served as the sacrificial electron donor in the system, and TEOA serves as a proton donor in the anhydrous system. The catalytic system obtained 18 mmol H_2_ after 7-h illumination [55]. Clearly, an additive sacrificial electron donor may improve the catalytic performance. Therefore, NHS was selected as an additive electron donor for the photocatalytic reaction in the present study. With the addition of NHS, other components showed similar control effects as that of the NHS-free system. To verify the potential proton donor in this dual sacrificial reagent system, an anhydrous system under otherwise identical conditions was tested and showed no H_2_ evolution (Figure 5a), indicating the important role of H_2_O in hydrogen generation. The optimal reaction condition was considered as 3 mg **1**, 0.060 M TEOA, 0.153 mM NHS, 2 M H_2_O, and 0.20 mM [Ir(ppy)_2_(dtbbpy)][PF_6_]. After 10-h photocatalysis, the H_2_ yield of 54.21 μmol was obtained, corresponding to the highest hydrogen evolution rate of 1807.07 μmol·g^−1^·h^−1^. A nine-fold increase in hydrogen yield was achieved by introducing NHS into the catalytic system compared to that of NHS-free catalytic system (Figure 5a). To better illustrate the strong catalytic performance of compound **1**, we have summarized the photocatalytic results of reported systems using cobalt-added tungsten-oxo clusters. Compared to the reported studies (Table S2), compound **1** exhibited one of the best photocatalytic performances in the absence of a noble metal co-catalyst (e.g., Pt). Also, to our knowledge, this is the first time that cobalt-added tungstogermanate has been investigated for visible-light-driven hydrogen production in the dual sacrificial reagent system. Considering that **1** contains Co^2+^, {GeW_9_O_34_} and {PO_4_}, additional control experiments using molar equivalents of Co(NO_3_)_2_·6H_2_O, K_8_Na_2_[A-α-GeW_9_O_34_], and Na_2_HPO_4_ as 3 mg of **1** have been further conducted to better understand the photocatalytic performance. As shown in Figure 5b, the systems catalyzed by Co(NO_3_)_2_·6H_2_O, K_8_Na_2_[A-α-GeW_9_O_34_], and Na_2_HPO_4_ resulted in the hydrogen yield of 6.32, 2.95, and 0.37 μmol, respectively. The mechanical mixture of all three components in equal molar proportions can catalyze the hydrogen production of 10.79 μmol (Figure 5b). All of these control experiment results demonstrated the importance of the intact molecular structure of **1** for effective photocatalysis.

In addition, it is worth mentioning that the color of the reaction system rapidly changed from bright yellow to dark blue upon light irradiation, simultaneously coupling the linear hydrogen evolution with the irradiation time. In the meanwhile, catalyst **1** was also transformed into the heteropoly blue intermediates, indicating the reduction of **1** during photocatalysis, which is commonly observed in the literature reports [42,54]. When the reaction system was moved to a dark environment, hydrogen production stopped immediately. The same phenomenon appeared in the light ON-OFF experiments (Figure 5c). After exposure to air, the heteropoly blue intermediates will return to their original pink color. The rapid formation and disappearance of heteropoly blue intermediates show the strong and reversible multielectron storage abilities of POMs. As discussed in previous reports, heteropoly blue intermediates act as an active species by receiving multiple electrons in the vacant W 5d orbitals, which promote the hydrogen evolution process [42,54]. The catalytic activity is also related to the amount of catalyst used. When the catalyst dosage was 1, 3, and 6 mg, the hydrogen yield reached 24.07, 54.21, and 51.44 μmol in 10 h, corresponding to the hydrogen production rate of 2407.62, 1807.07, and 857.46 μmol·g^−1^·h^−1^, respectively (Figure 5d and Figure S7). In addition, the concentration of photosensitizers is also a significant factor to the enhancement of hydrogen production. When the concentration of [Ir(ppy)_2_(dtbbpy)][PF_6_] was changed from 0.10 to 0.25 mM, the hydrogen evolution exhibited linear growth with reaction time, yielding 11.04 to 71.84 μmol H_2_ after 10-h. The hydrogen evolution rates ranged from 924.47 to 2394.91 μmol·g^−1^·h^−1^ (Figure 5e). Moreover, because the experiments varied the concentration of TEOA from 0.03 to 0.06 M, the hydrogen production ranged from 35.72 to 54.21 μmol. The calculated hydrogen evolution rate ranged from 1190.69 to 1807.07 μmol·g^−1^·h^−1^ (Figure 5f). It is noted that varying the concentration of NHS from 0.075 mM to 0.300 mM barely changed the H_2_ yield, which maintained a constant concentration of TEOA (0.060 M) (Figure S8).

**Figure 6:**
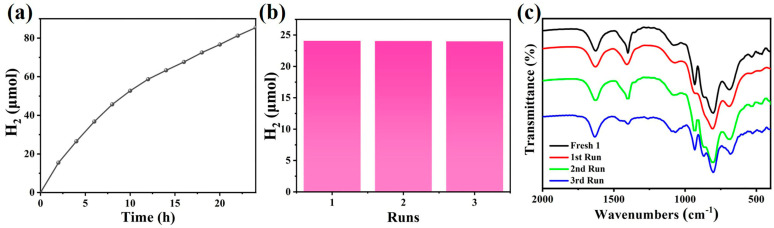
(**a**) Long term photocatalysis of water reduction reaction test; (**b**) Recyclability tests of **1** towards hydrogen generation in three cycles. (Reaction conditions: 10 W white LED light, **1** (1 mg), TEOA (0.060 M), NHS (0.153 mM), [Ir(ppy)_2_(dtbbpy)][PF_6_] (0.2 mM), H_2_O (2 M), CH_3_CN/DMF (1/3) as mixed solvent, the total reaction volume of 6 mL, stirring speed 400 rpm, at room temperature); (**c**) FT−IR spectra of fresh and the isolated **1** after the catalytic cycles.

In order to further assess the stability of **1** in the photocatalytic process, a long-term reaction lasting 24 h was carried out, and an amount of 85.40 μmol of hydrogen was cumulatively produced (Figure 6a). The hydrogen production rate gradually decreased over time due to the partial consumption of the sacrificial reagent and photosensitizer in the catalytic system. The catalysts after photocatalysis were isolated by centrifugation and washed with CH_3_CN. The isolated catalyst was further used for three successive photocatalytic cycles in the presence of fresh sacrificial reagents and the photosensitizer; the photocatalytic hydrogen production performance of **1** was maintained with negligible decline of the H_2_ yield (Figure 6b), proving the good stability and recycling robustness of catalyst **1**. The structural stability of catalyst **1** was further confirmed by the perfectly matched FT-IR spectra of the isolated catalyst with that of the fresh compound **1** (Figure 6c). In order to prove the heterogeneity of the reaction system, an experiment was carried out to analyze the leaching of Co in the post-catalysis solution using the ICP-OES test. The results showed there is little Co (0.007 μmol) dissolution after the photocatalytic reaction (Table S3), which cannot significantly contribute to the hydrogen production activity, as demonstrated by the control experiment (Figure 5b, red line). Overall, all above experimental results and analyses demonstrated that compound **1** can work as a promising heterogeneous catalyst for visible-light-driven hydrogen production with decent activity, stability, and recyclability.

## 4. Conclusions

Under hydrothermal conditions, a water-insoluble and stable CoAPs, H_31_(NH_4_)_5_-Na_16_{Co^Ⅲ^(H_2_O)_6_}_4_{[Co^Ⅱ^_4_(μ_3_-OH)_3_(PO_4_)]_4_(A-*α*-GeW_9_O_34_)_4_}_2_·23H_2_O (**1**), was synthesized and characterized by various techniques. Distinguished from the reported hexadecanuclear cobalt-added POM, four additional free {Co(H_2_O)_6_} crystallized in **1**. The introduction of phosphate linkers contributes to the aggregation of cobalt-oxo clusters. The hydrothermal synthetic method is beneficial for the synthesis of stable and structurally-new compound **1**. While coupling with the photosensitizer and sacrificial reagent, **1** worked as a heterogeneous catalyst for visible-light-driven hydrogen evolution. Under optimal catalytic conditions, a hydrogen yield of 54.21 μmol was achieved after 10-h photocatalysis, corresponding to a hydrogen evolution rate of 1807.07 μmol·g^−1^·h^−1^. Compared to the classic three-component HER system, NHS was firstly applied to construct a dual sacrificial reagent system, which can significantly enhance the hydrogen evolution activity. The photocatalytic results confirm the enhanced effects of such dual-electron-sacrificial-reagents systems. This study provides new guidance for designing highly stable cobalt-added POMs and expanding their catalytic applications in solar conversion.

## Figures and Tables

**Table 1:** Crystallographic data and structure refinement for **1**.

	1
Empirical formula	H_169_N_5_O_375_Na_16_P_8_Co_36_Ge_8_W_72_
Formula weight	22,794.97
Temperature/K	293(2)
Crystal system	Triclinic
Space group	*P*-1
a/Å	24.4950 (8)
b/Å	30.9701 (17)
c/Å	36.8329 (17)
α/°	72.049 (4)
β/°	70.58
γ/°	66.70
Volume/Å^3^	23,694.9 (19)
Z	2
ρ_calc_g/cm^3^	3.113
μ/mm^−1^	19.239
F(000)	19,358.0
Goodness-of-fit on F^2^	0.900
Final R indexes [I ≥ 2σ (I)] ^a,b^	*R*_1_ = 0.0643, w*R*_2_ = 0.1158
Final R indexes [all data] ^a,b^	*R*_1_ = 0.1428, w*R*_2_ = 0.1420

^a^ *R*_1_ = ∑||*F*o| − |*F*c||/∑|*F*o|; ^b^ w*R*_2_ = [Σ*w*(*F*o^2^ − *F*c^2^)^2^]/Σ*w*(*F*o^2^)^2^]^1/2^.

## Data Availability

The data presented in this study are available on request from the corresponding author.

## Supplementary Materials

The following supporting information can be downloaded at: https://www.mdpi.com/article/10.3390/nano13132009/s1, Figure S1. TGA curve of **1**. Figure S2. Ball and stick model diagram of **1**. Color codes: W atom, black; Ge atom, orange; Co atom, turquoise; P atom, yellow; O atom, red. Figure S3. The packing mode of **1** in the *bc* plane. Color codes: WO_6_, red; CoO_6_, turquoise; PO_4_, yellow; GeO_4_, orange. Figure S4. FT-IR spectrum of **1**. Figure S5. Experimental and simulated PXRD patterns of **1**. Figure S6. UV-Vis diffuse reflectance spectrum of **1** and the corresponding α/S-Energy curve (inset). Figure S7. Relationship between hydrogen yield and hydrogen evolution rate in different qualities of **1**. Figure S8. Hydrogen production comparison of different concentrations of NHS. Table S1. Control experiments for photocatalytic water reduction reaction in NHS-free conditions. Table S2. Overview of different photocatalytic HER performance using cobalt-added tungsten-oxo clusters [39,40,41,56,57]. Table S3. ICP-OES analyses of the cobalt contents in compound **1** and the post-catalytic solution after 10-h photocatalysis.
