# Supplementary material for: A Hexadecanuclear Cobalt-Added Tungstogermanate Containing Counter Cobalt Hydrates: Synthesis, Structure and Photocatalytic Properties
Source: Nanomaterials (Basel). 2023 Jul 5;13(13):2009. doi: 10.3390/nano13132009 (PMC10343794; doi:10.3390/nano13132009)
Supplement: Supplementary file 1 [file nanomaterials-13-02009-s001.zip › nanomaterials-2472219-supplementary.pdf]

nanomaterials

MDPI

Supplementary Material

A Hexadecanuclear Cobalt-Added Tungstogermanate Containing Counter Cobalt Hydrates: Synthesis, Structure and Photocatalytic Properties

Qing Zhao, Xuyan Li, Yu Wang, Hongjin Lv * and Guoyu Yang *

MOE Key Laboratory of Cluster Science, School of Chemistry and Chemical Engineering, Beijing Institute of Technology, Beijing 102488, China; zhaoqing980121@163.com (Q.Z.); lxy_bitchem@163.com (X.L.); yuwang0414@outlook.com (Y.W.)
* Correspondence: hlv@bit.edu.cn (H.L.); ygy@bit.edu.cn (G.Y.)

Table of Contents

Figure S1. TGA curve of **1**.
Figure S2. Ball and stick model diagram of **1**. Color codes: W atom, black; Ge atom, orange; Co atom, turquoise; P atom, yellow; O atom, red.
Figure S3. The packing mode of **1** in *bc* plane. Color codes: WO_6, red; CoO_6, turquoise; PO_4, yellow; GeO_4, orange.
Figure S4. FT-IR spectrum of **1**.
Figure S5. Experimental and simulated PXRD patterns of **1**.
Figure S6. UV-Vis diffuse reflectance spectrum of **1** and the corresponding α/S-Energy curve (inset).
Figure S7. Relationship between hydrogen yield and hydrogen evolution rate in different qualities of **1**.
Figure S8. Hydrogen production comparison of different concentrations of NHS.
Table S1. Control experiments for photocatalytic water reduction reaction in NHS-free conditions..
Table S2. Overview of different photocatalytic HER performance using cobalt-added tungsten-oxo clusters.
Table S3. ICP-OES analyses of the cobalt contents in compound **1** and the post-catalytic solution after 10-hours photocatalysis.

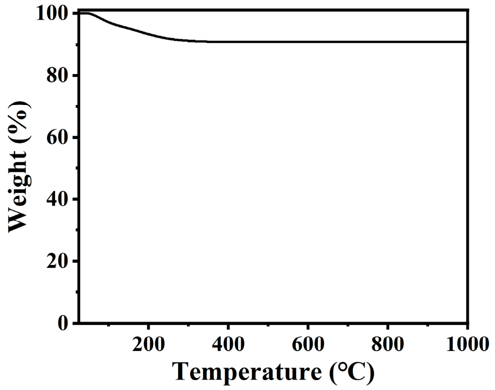

Figure S1. TGA curve of **1**.

As shown in Figure S1, the weight loss occurs in one step from 49.5 to 350.83 °C. The total loss ratio of 9.08% corresponds to the removal of 36 adsorbed water molecules, 23 crystallized water molecules, 24 coordinated water molecules, 5 NH_4^+, 24 OH, and 31 protons.

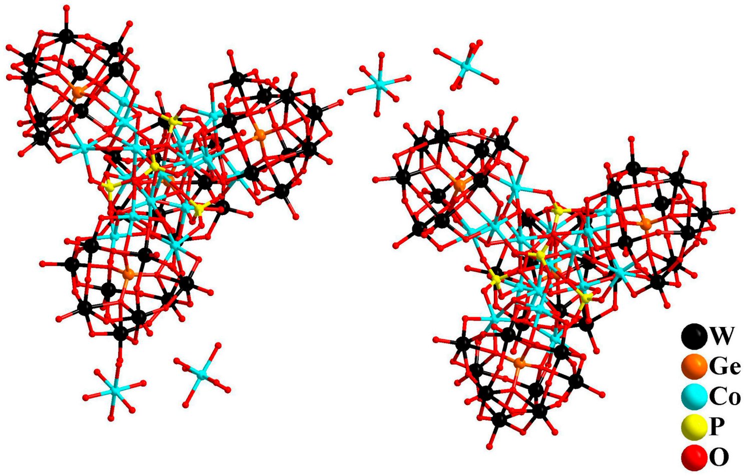

Figure S2. Ball and stick model diagram of **1**. Color codes: W atom, black; Ge atom, orange; Co atom, turquoise; P atom, yellow; O atom, red.

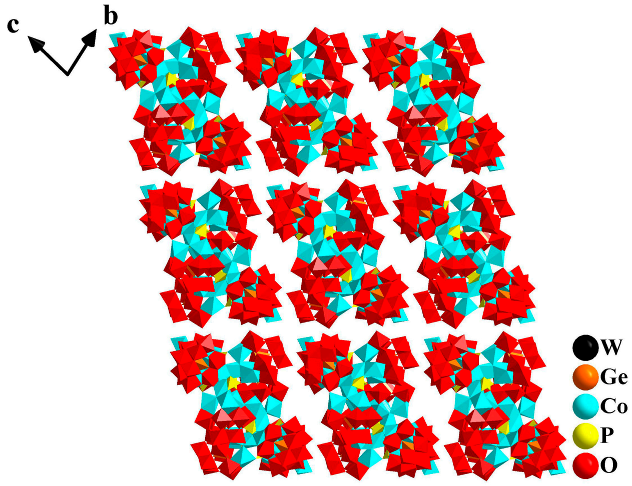

Figure S3. The packing mode of **1** in *bc* plane. Color codes: WO_6, red; CoO_6, turquoise; PO_4, yellow; GeO_4, orange.

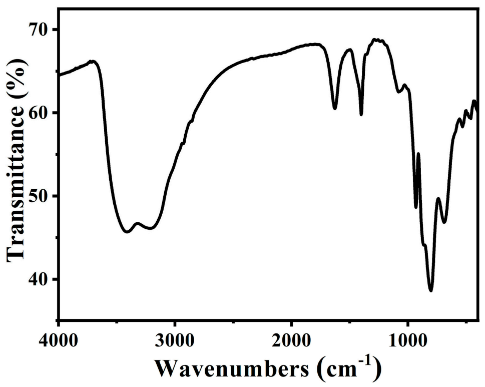

Figure S4. FT-IR spectrum of **1**.

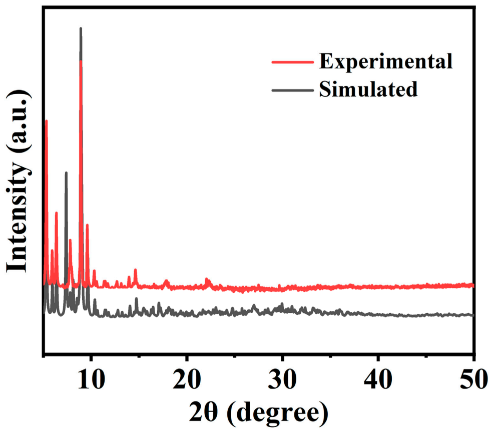

Figure S5. Experimental and simulated PXRD patterns of **1**.

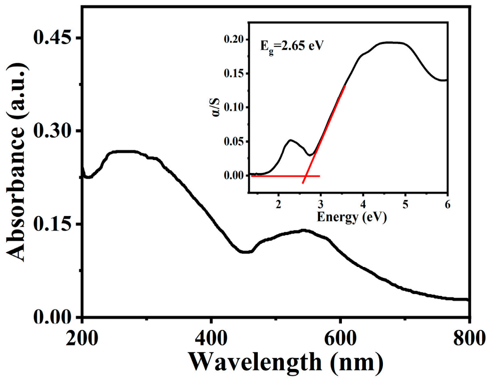

Figure S6. UV-Vis diffuse reflectance spectrum of **1** and the corresponding α/S-Energy curve (inset).

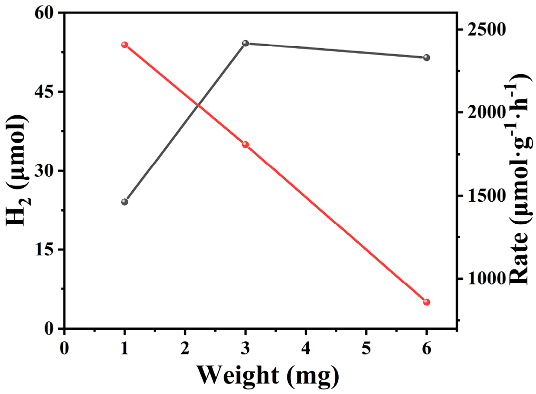

Figure S7. Relationship between hydrogen yield and hydrogen evolution rate in different qualities of **1** (Reaction conditions: 10 W white LED light, **1** (1, 3, 6 mg), TEOA (0.060 M), NHS (0.153 mM), $[Ir(ppy)_2(dtbb\text{-}py)][PF_6]$ (0.2 mM), H_2O (2 M), CH_3CN/DMF (1/3) as mixed solvent, the total reaction volume of 6 mL, stirring speed 400 rpm, at room temperature).

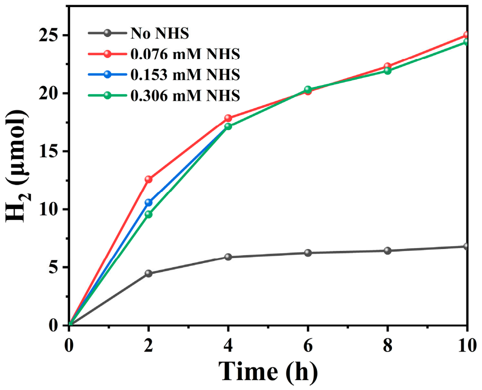

Figure S8. Hydrogen production comparison of different concentrations of NHS (Reaction conditions: 10 W white LED light, **1** (1 mg), TEOA (0.060 M), NHS (0.076, 0.153, 0.306 mM), $[Ir(ppy)_2\text{-}(dtbbpy)][PF_6]$ (0.2 mM), H_2O (2 M), CH_3CN/DMF (1/3) as mixed solvent, the total reaction volume of 6 mL, stirring speed 400 rpm, at room temperature).

Table S1. Control experiments for photocatalytic water reduction reaction in NHS-free conditions.

Conditions	H_2 yield (μmol)
TEOA + PS	0.42
Catalyst + PS	0
Catalyst + TEOA	0

Table S2. Overview of different photocatalytic HER performance using cobalt-added tungsten-oxo clusters.

Catalyst	Light source	Photosensitizer	Sacrificial agent	cocatalyst	H_2 generation rate	Ref
{$CoSiW_{11}$}	500 W Xe lamp	None	Zn powder	Pt	65 $\mu mol \cdot g^{-1} \cdot h^{-1}$	1
{$CoTiW_{11}$}	simulated solar light (350–760 nm)	None	polyvinyl alcohol	None	484 $\mu mol \cdot g^{-1} \cdot h^{-1}$	2
{$CoCoW_{11}$}	3 W LED light	Eosin Y	TEOA	Pt	13395 $\mu mol \cdot g^{-1} \cdot h^{-1}$	3
{Co_6P_3}	300 W Xe lamp	Eosin Y	TEOA	None	141 $\mu mol \cdot h^{-1}$	4
{Co_9P_3}	10 W white LED light	[Ir(ppy)$_2$(dtbbpy)][PF_6]	TEOA	None	1217.6 $\mu mol \cdot g^{-1} \cdot h^{-1}$	5
**{$Co_{16}Ge_4$}**	10 Wwhite LED light	[Ir(ppy)$_2$(dtbbpy)][PF_6]	TEOA and NHS	none	1807.07 $\mu mol \cdot g^{-1} \cdot h^{-1}$	**This work**

Table S3. ICP-OES analyses of the cobalt contents in compound **1** and the post-catalytic solution after 10-hours photocatalysis.

Reaction time (h)	Concentration of Co in **1** for photocatalysis (μmol)	Concentration of Co in the post-catalysis solution (μmol)
10	4.738	0.007

References

1. Wang, Z.-L.; Lu, Y.; Li, Y.-G.; Wang, S.-M.; Wang, E.-B. Visible-light photocatalytic H_2 evolution over a series of transition metal substituted Keggin-structure heteropoly blues. *Chinese Science Bulletin* **2012**, *57*, 2265-2268. https://doi.org/10.1007/s11434-012-5050-1
2. Shang, X.-K.; Liu, R.-J.; Zhang, G.-J.; Zhang, S.-J.; Cao, H.-B.; Gu, Z.-J. Artificial photosynthesis for solar hydrogen generation over transition-metal substituted Keggin-type titanium tungstate. *New J. Chem.* **2014**, *38*, 1315-1320. https://doi.org/10.1039/C3NJ01184D
3. Zhao, J.-L.; Ding, Y.; Wei, J.; Du, X.-Q.; Yu, Y.-Z.; Han, R.-X. A Molecular Keggin Polyoxometalate Catalyst with High Efficiency for Visible-light Driven Hydrogen Evolution. Int. J. Hydrogen Energy. 2014, 39, 18908–18918. https://doi.org/10.1016/j.ijhydene.2014.09.084
4. Wu, W.-M.; Teng, T.; Wu, X.-Y.; Dui, X.-J.; Zhang, L.; Xiong, J.-H.; Wu, L.; Lu, C.-Z. A Cobalt-based Polyoxometalate Catalyst for Efficient Visible-light-driven H_2 Evolution from Water Splitting. *Catal. Commun.* **2015**, *64*, 44–47. https://doi.org/10.1016/j.catcom.2015.01.032
5. Wang, Z.-W.; Yang, G.-Y. A {Co_9}-Added Polyoxometalate for Efficient Visible-light-driven Hydrogen Evolution. *Molecules.* **2023**, *28*, 664. https://doi.org/10.3390/molecules28020664
